# Identification of an Antimicrobial Peptide from the Venom of the Trinidad Thick-Tailed Scorpion *Tityus trinitatis* with Potent Activity against ESKAPE Pathogens and *Clostridioides difficile*

**DOI:** 10.3390/antibiotics12091404

**Published:** 2023-09-04

**Authors:** Milena Mechkarska, Taylor S. Cunning, Megan G. Taggart, Nigel G. Ternan, Jérôme Leprince, Laurent Coquet, Thierry Jouenne, Jordi Tena-Garcés, Juan J. Calvete, J. Michael Conlon

**Affiliations:** 1 Department of Life Sciences, Faculty of Science and Technology, St. Augustine Campus, The University of The West Indies, St. Augustine, Trinidad and Tobago; 2 Nutrition Innovation Centre for Food and Health (NICHE), School of Biomedical Sciences, Ulster University, Coleraine BT52 1SA, UK; cunning-t@ulster.ac.uk (T.S.C.); m.taggart1@ulster.ac.uk (M.G.T.); ng.ternan@ulster.ac.uk (N.G.T.); 3 Université Rouen Normandie, INSERM, NorDiC UMR 1239, HeRacLeS, US 51, PRIMACEN, F-76000 Rouen, France; jerome.leprince@univ-rouen.fr; 4 Université Rouen Normandie, INSA Rouen Normandie, CNRS, PBS UMR 6270, HeRacLeS US 51 UAR 2026 PISSARO, F-76000 Rouen, France; laurent.coquet@univ-rouen.fr (L.C.); thierry.jouenne@univ-rouen.fr (T.J.); 5 Evolutionary and Translational Venomics Laboratory, Consejo Superior de Investigaciones Científicas (CSIC), 46010 Valencia, Spain; jtena@ibv.csic.es (J.T.-G.); jcalvete@ibv.csic.es (J.J.C.); 6 Diabetes Research Centre, School of Biomedical Sciences, Ulster University, Coleraine BT52 1SA, UK; m.conlon@ulster.ac.uk

**Keywords:** scorpion venom, ESKAPE pathogen, *Clostridioides difficile*, antimicrobial peptide, neurotoxin

## Abstract

Envenomation by the Trinidad thick-tailed scorpion *Tityus trinitatis* may result in fatal myocarditis and there is a high incidence of acute pancreatitis among survivors. Peptidomic analysis (reversed-phase HPLC followed by MALDI-TOF mass spectrometry and automated Edman degradation) of *T. trinitatis* venom led to the isolation and characterization of three peptides with antimicrobial activity. Their primary structures were established asTtAP-1 (FLGSLFSIGSKLLPGVFKLFSRKKQ.NH2), TtAP-2 (IFGMIPGLIGGLISAFK.NH2) and TtAP-3 (FFSLIPSLIGGLVSAIK.NH2). In addition, potassium channel and sodium channel toxins, present in the venom in high abundance, were identified by CID-MS/MS sequence analysis. TtAP-1 was the most potent against a range of clinically relevant Gram-positive and Gram-negative aerobes and against the anaerobe *Clostridioides difficile* (MIC = 3.1–12.5 µg/mL). At a concentration of 1× MIC, TtAP-1 produced rapid cell death (<15 min against *Acinetobacter baumannii* and *Staphylococcus aureus*). The therapeutic potential of TtAP-1 as an anti-infective agent is limited by its high hemolytic activity (LC_50_ = 18 µg/mL against mouse erythrocytes) but the peptide constitutes a template for the design of analogs that maintain the high bactericidal activity against ESKAPE pathogens but are less toxic to human cells. It is suggested that the antimicrobial peptides in the scorpion venom facilitate the action of the neurotoxins by increasing the membrane permeability of cells from either prey or predator.

## 1. Introduction

Scorpion venom has proved to be a rich source of biologically active peptides that may have therapeutic potential. These include compounds with antimicrobial, anti-tumor, anti-inflammatory, anti-angiogenic and analgesic properties, and peptides that may be developed into drugs for treatments of diseases associated with ion channels (reviewed in [1]). The genus *Tityus*, belonging to the extensive family Buthidae, comprises more than 200 species that are widely distributed in Central and South America. Venoms from these scorpions have been extensively investigated for the presence of clinically relevant peptides (reviewed in [2]). *Tityus* species whose venoms contain peptides with antimicrobial activity include *Tityus asthenes* [3], *Tityus bahiensis* [4], *Tityus costatus* [5], *Tityus discrepans* [6], *Tityus obscurus* [7], *Tityus serrulatus* [7,8,9] and *Tityus stigmurus* [10,11,12]. The most intensively studied antimicrobial peptides (AMPs) from *Tityus* species are stigmurin and TsAP-2 from *T. stigmurus,* which display broad spectrum bactericidal activities and antiproliferative effects on tumor cells in vitro and reduce leukocyte migration, TNF-α levels, microorganism load in the peritoneal cavity and inflammation in the lung and cecum in a murine model of polymicrobial sepsis [12]. In addition, serrulin, a glycine-rich peptide from *T. serrulatus*, shows both antibacterial and antifungal activities [13], and potent activity against a range of *Candida* species was shown by ToAP-2 from *T. obscurus* [14].

The Trinidad thick-tailed scorpion *Tityus trinitatis* is endemic to Trinidad and Tobago and has a limited distribution in Venezuela [15]. The species is of clinicopathological importance as envenomation may result in myocarditis and coagulative myocytolysis and also a failure of the respiratory system that may be fatal in the cases of the very young and the elderly and infirm [16]. In the case of non-lethal envenomation, there is a high incidence of acute pancreatitis among survivors [17]. Intravenous injection of crude *T. trinitatis* venom into fasting anesthetized dogs stimulated secretion of the exocrine pancreas [18] and the release of insulin [19]. The venom stimulated amylase release from rat pancreatic slices by a cholinergic mechanism that may involve muscarinic receptors [20].

The ever-increasing emergence of pathogenic microorganisms with resistance to commonly used antibiotics constitutes a major public health crisis and necessitates a search for new types of therapeutic agents [21]. The aim of the present study was to use the method of peptidomic analysis (reversed-phase HPLC followed by MALDI-TOF mass spectrometry and automated Edman degradation) to identify and purify peptides with antibacterial activity in electrically stimulated samples of venom from female *T. trinitatis*. The therapeutic potential of synthetic replicates of the peptides as anti-infective agents was evaluated by determining their growth-inhibitory activity against a range of clinically relevant bacteria with a focus on ESKAPE pathogens and *Clostridioides difficile*. The term ESKAPE refers to a group of highly virulent and antibiotic-resistant nosocomial pathogens comprising *Enterococcus faecium*, *Staphylococcus aureus*, *Klebsiella pneumoniae*, *Acinetobacter baumannii*, *Pseudomonas aeruginosa* and *Enterobacter* spp. (sometimes extended to include *Escherichia coli*) that can “escape” the effect of the commonly used antibiotics due to their ability to develop multi-drug resistance [22,23].

## 2. Results

### 2.1. Purification of the Peptides

**Figure 1:**
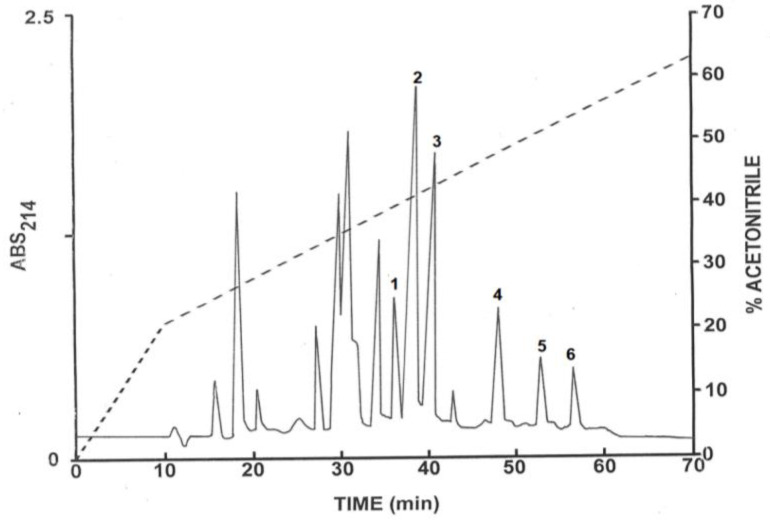
Reversed-phase HPLC on a semipreparative Vydac C-18 column of venom from female *T. trinitatis* after partial purification on Sep-Pak C-18 cartridges. The dashed line shows the concentration of acetonitrile in the eluting solvent. The peaks denoted 1–3 contained peptides in major abundance with molecular masses in the range 5000 to 8000 Da. The peaks denoted 4–6 contained peptides with antimicrobial activity. The peptides in peaks 1–6 were purified further.

**Figure 2:**
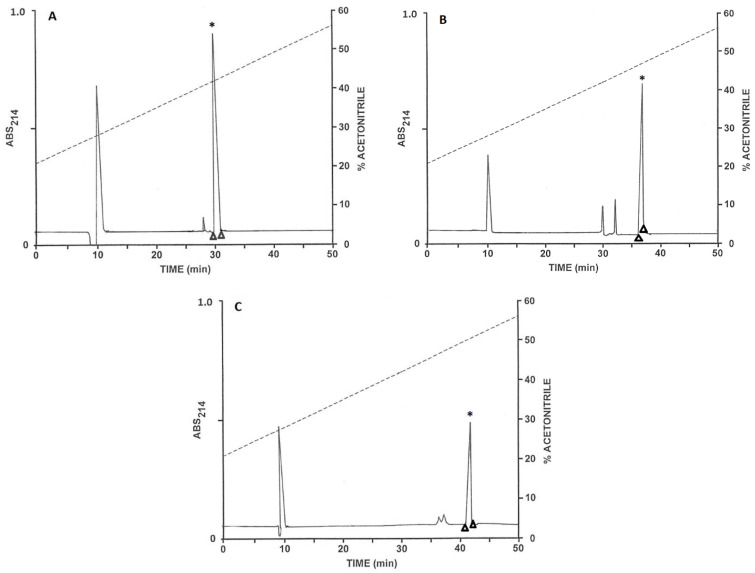
Purification to near homogeneity of (**A**) TtAP-1, (**B**) TtAP-2 and (**C**) Tt-AP3 on a semipreparative Vydac C-4 column. The triangles show where peak collection began and ended. * denotes the peak containing the antimicrobial peptide. The dashed line shows the concentration of acetonitrile in the eluting solvent.

The pooled samples of venom, after partial purification on Sep-Pak C-18 cartridges, were chromatographed on a Vydac C-18 semipreparative reversed-phase HPLC column (Figure 1). The prominent peaks with retention times between 35 and 56 min were collected and aliquots (20 µL) were analysed by MALDI-TOF mass spectrometry. The peptides in major abundance in peaks 1–3 (Figure 1) had molecular masses in the range 5000 to 8000 Da and were further purified on a semipreparative Vydac C-4 column as described in Supplementary Materials (chromatograms not shown). The peaks designated 4–6 (Figure 1), which displayed antibacterial activity, were subjected to further purification on a semipreparative Vydac C-4 column (Figure 2). Subsequent structural analysis showed that peak 4 contained TtAP-1, peak 5 TtAP-2 and peak 6 TtAP-3. The peptides were obtained in near homogeneous form (purity > 98%), as assessed by a symmetrical peak shape and mass spectrometry.

### 2.2. Identification of the Sodium Channel and Potassium Channel Toxins

The identities of the peptides present in peaks 1–3 (Figure 1) were determined by collision-induced dissociation (CID)-MS/MS mass spectrometric analysis of fragments generated by in-gel trypsin digestion [24]. The tryptic peptides KDGYIIEHR (KDGYIIEHR), YSCFFGTNTWCNTECTXK (YSCFFGTNSWCNTECTLK) and CWCYGXPNDVK (CWCWGLPDNVK) generated from the major component in peak 1 identified it as the homolog of the sodium channel toxin TdNa5 from *T. discrepans*. The residues in parentheses are the corresponding tryptic fragments from *T. discrepans*. The tryptic fragments SEYACPVIDKFCEDHCAAK (SEYACPVIDKFCEDHCAAK) and SEYACPVIDK (SEYACPVIDK) identified a major component in peak 2 as the homolog of the potassium channel toxin TdKIK from *T. discrepans*. The tryptic fragments DAYPANWR (DAYPADWR), CWCYGXPDWR (CWCYGLPDSVR), and RDAYPANWR (RDAYPADWR) identified a second major component in peak 2 as the homolog of the sodium channel toxin TdNa1 from *T. discrepans*. The tryptic fragments TQFGCPAYEGYCMNHCQDXER (TQFGCPAYEGYCMNHCQDIER) and NEGXCHGFK (HDGSCHGFK) identified the major component in peak 3 as the homolog of the potassium channel toxin Tdi-β-KTx from *T. discrepans* [6,25]. These data together with the observed molecular masses of the toxins and UniProtKB/Swiss-Prot accession numbers of the *T. discrepans* toxins are summarized in Table 1.

### 2.3. Structural Characterization of the Antimicrobial Peptides

The primary structures of TtAP-1, TtAP-2 and TtAP-3 were established without ambiguity by automated Edman degradation, and their complete primary structures are shown in Table 2. The molecular masses of the peptides, determined by MALDI-TOF mass spectrometry, were consistent with the proposed structures and are also shown in Table 2. The data indicated that the three peptides are C-terminally α-amidated. The calculated physicochemical properties of TtAP-1, TtAP-2 and TtAP-3 are shown in Table 3.

### 2.4. Antimicrobial and Hemolytic Activities

**Figure 3:**
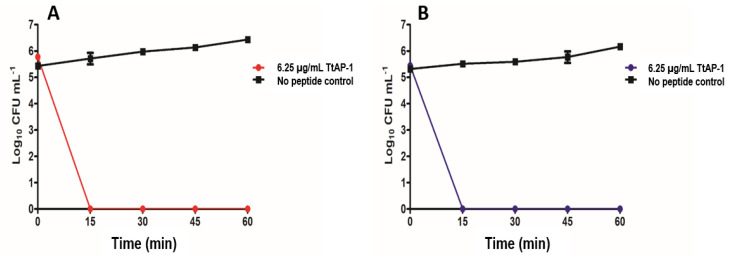
Survival of *A. baumannii* (DSM 30008) (**A**) and *S. aureus* (ATCC 43300) (**B**) in Mueller–Hinton broth after the addition of 6.25 µg/mL TtAP-1 (equal to 1× MIC). Control incubations were carried out in the absence of peptide.

The growth inhibitory activities of synthetic replicates of TtAP-1, TtAP-2 and TtAP-3 against reference strains of the ESKAPE pathogens (*E. faecium*, *S. aureus*, *K. pneumoniae*, *A. baumannii*, *P. aeruginosa* and *E. coli*) together with *Enterococcus faecalis*, *Staphylococcus epidermidis* and *C. difficile* are shown in Table 4. The table also shows corresponding minimum inhibitory concentrations (MICs) for gentamicin and vancomycin in control incubations. Time-kill assays using *A. baumannii* and *S. aureus* were performed with TtAP-1 at a concentration of 6.25 µg/mL (equal to 1× MIC). The data indicate rapid and effective antimicrobial action with the killing of >99.9% of both microorganisms within 15 min (Figure 3).

The hemolytic activities (LC_50_) of synthetic replicates of the scorpion peptides against mouse erythrocytes were TtAP-1 18 ± 2 µg/mL, TtAP-2 31 ± 4 µg/mL, TtAP-3 95 ± 3 µg/mL.

## 3. Discussion

**Figure 4:**
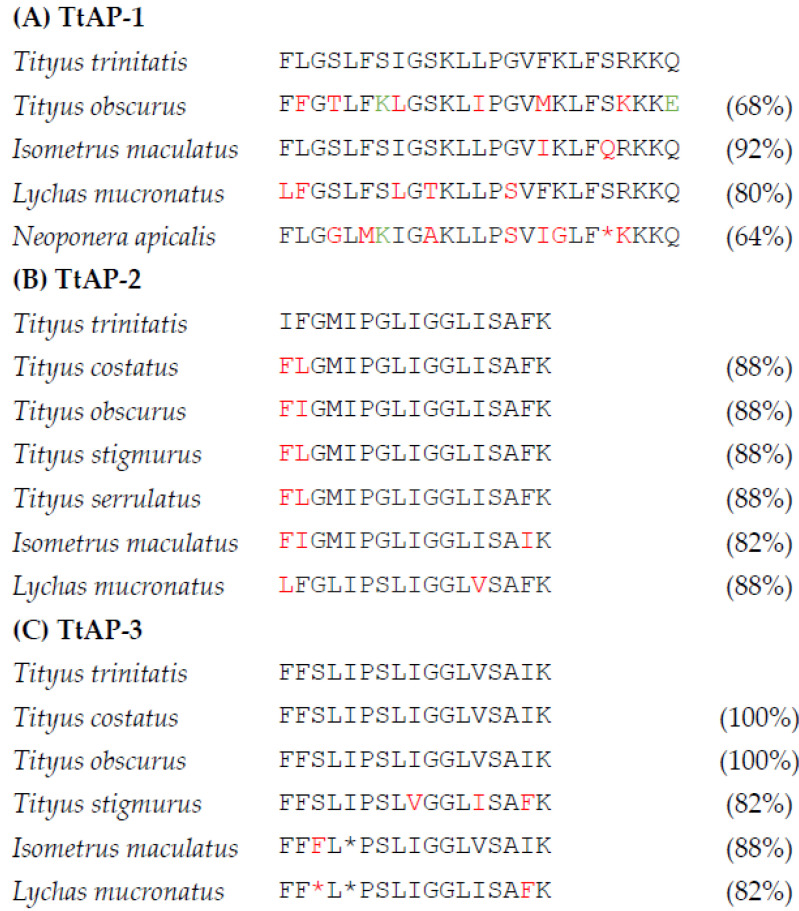
A comparison of the primary structures of (**A**) TtAP-1 with structurally similar peptides from other scorpion species and poneratoxin-Na1a from the ant *N. apicalis*, (**B**) TtAP-2 with peptides similar to TsAP-2 from other scorpion species and (**C**) TtAP-3 with peptides similar to stigmurin from other scorpion species. Conservative amino acid substitutions are shown in red and non-conservative substitutions in green. Gaps denoted by * have been inserted in some sequences to maximize structural similarity. The numbers in parentheses indicate the % identity with each of the *T. trinitatis* peptides, respectively.

The study has led to the purification and identification of three peptides present in high abundance in the venom of the scorpion with varying degrees of antibacterial and hemolytic activities. As shown in Figure 4, TtAP-1 shows structural similarity with peptides previously isolated from the venoms of the Amazonian black scorpion *T. obscurus* [7], the lesser brown scorpion *Isometrus maculatus* [30], the Chinese swimming scorpion *Lychas mucronatus* [31,32] and unexpectedly with poneratoxin-Na1a from the stinging ant *Neoponera apicalis* [33]. Like TtAP-1, poneratoxin-Na1a has broad spectrum of activity against both Gram-positive and Gram-negative bacteria and displays strong hemolytic activity [33]. TtAP-2 is structurally similar to TsAP-2 isolated from the venoms of a range of *Tityus* species [5,7,8,12] and also from *I. maculatus* [30] and *L. mucronatus* [32] venoms. TtAP-3 is identical to stigmurin from *T. costatus* [5] and *T. obscurus* [7] and structurally similar to the peptides from *T. stigmurus* [11] and to imcoporin from *I. maculatus* [30] and mucroporin from *L. mucronatus* [32].

The most important result arising from the study is the observation that TtAP-1 shows potent activity against the clinically relevant Gram-positive and Gram-negative ESKAPE^+^ pathogens (MICs in the range 6–12 µg/mL equivalent to approx. 2–4 µM) with particular high potency against the anaerobe *C. difficile* (MIC = 3 µg/mL equivalent to approx. 1 µM) (Table 4). The mechanism of action of the peptide was not investigated in this study but the very rapid rate of killing of *A. baumannii* and *S. aureus* (Figure 3) strongly suggests that the peptide is disrupting the integrity of the bacterial cell membrane rather than inducing apoptosis or inhibiting peptide synthesis by binding to DNA. The relative potencies of helical peptides against bacteria and fungi and the cytolytic activities against mammalian cells, such as erythrocytes, are determined by complex interactions between cationicity, hydrophobicity, conformation (stability and extent of the helix) and amphipathicity [34]. As shown in Table 3, TtAP-1, TtAP-2 and TtAP-3 are strongly hydrophobic and have the predicted propensity to adopt helical conformations over a region of their structures.

**Figure 5:**
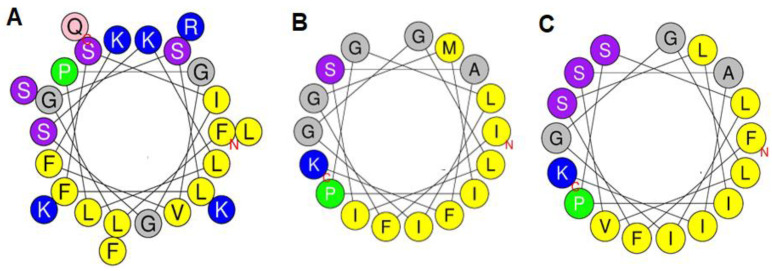
A Schiffer–Edmundson wheel representation of the conformation of (**A**) TtAP-1, (**B**) TtAP-2 and (**C**) TtAP-3. Basic amino acids are shown in blue and strongly hydrophobic amino acids are shown in yellow. The other colours show non-charged residues with varying degrees of hydrophilicity.

In the case of TtAP-3-related peptide stigmurin from *T. stigmurus*, the prediction has been confirmed by NMR studies in trifluoroethanol:water mixtures with the peptide exhibiting a random conformation at the N-terminus region (Phe^1^ to Pro^6^) and a helical structure from Ser^7^ to Phe^16^ [35]. Consistent with their calculated hydrophobic moments, a helical wheel representation of the three peptides demonstrates that they possess strongly amphipathic conformations with the hydrophobic Phe, Leu, Ile and Val residues segregating together on one face of the helix and charged residues on the opposite face (Figure 5). The appreciable higher antibacterial potency of TtAP-1 is probably a consequence of its greater positive charge (+6 compared with +2 for TtAP-2 and TtAP-3 at pH 7). Studies with analogs of a wide range of cell-penetrating α-helical peptides isolated from frog skin secretions have shown that increasing positive charge on the peptides results in an increase in antibacterial activity until a limit is reached whereupon further increases in cationicity do not increase and may decrease activity. However, increasing cationicity without concomitant changes in conformation generally results in increased hemolytic activity [36,37]. Similar observations have been reported for analogs of antimicrobial peptides from scorpions [38]. This effect is consistent with the observed greater hemolytic activity of TtAP-1 compared with TtAP-2 and TtAP-3.

The indiscriminate use of antibiotics has resulted in the emergence of multidrug-resistant (MDR) and extensively drug-resistant (XDR) bacteria, against which even the last line of defense antimicrobials, such as colistin, are ineffective [21,22,23]. A report commissioned by the UK government noted that roughly 700,000 people die annually from infections caused by MDR and XDR bacteria, a figure projected to increase to 10 million deaths per year by 2050 [21]. Consequently, new antimicrobials are desperately needed, and natural products, such as compounds from plants [39,40], amphibian skin secretions [41], and snake venoms [42], represent a potentially underutilized resource in this regard. This study has identified a peptide present in scorpion venom (TtAP-1) with high potency against a range of clinically relevant Gram-positive and Gram-negative bacteria including ESKAPE pathogens (Table 4). Of particular note is the very high potency against two strains of *C. difficile*. This bacillus is a Gram-positive spore-forming, toxigenic anaerobe that causes recurrent diarrhea in patients with compromised gut microbiota, which can progress to life-threatening inflammation of the colon. Thus, it has been recognized as the most common cause of healthcare-associated infection (HAI) globally [43]. Consequently, in 2019 it was ranked an “urgent threat” by the Centers for Disease Control and Prevention (CDC) [44].

As well as isolating antibacterial peptides in *T. tityus* venom, the study has identified two sodium channel toxins on the basis of structural similarity with toxins TdNa1 and TdNa5, isolated from *T. discrepans* venom. These toxins block voltage-gated sodium (Nav) channels in an irreversible manner. They are selective for arthropods but are non-toxic to mice [25,45]. In addition, two potassium channel toxins were identified on the basis of structural similarity with toxins Tdi-beta-KTx and TdKIK from *T. discrepans*. These toxins blocked voltage-gated (Kav) potassium channels [25,45]. Full structural characterization of these ion-channel toxins and investigation of their biological activities will be the subject of future research.

The biological role of AMPs in scorpion venom is unclear and they may be multifunctional. In the case of amphibians, such peptides are released in secretions that bathe the skin and serve to protect the organism against the over-growth of pathogenic microorganisms encountered in the environment. Peptides present only in venom are unlikely to play such a role. However, it has been proposed that the cell-penetrating AMPs in frog skin secretions may have the additional defense function of facilitating the action of toxins in the secretions by increasing membrane permeability in the cells of predators following attempted ingestion [46]. The venoms of scorpions of the genus *Tityus* contain a wide range of neurotoxins so that highly cytotoxic peptides such as TtAP-1 may play a similar role in not only protecting against predators but also facilitating rapid immobilization of prey. Another possible function of AMPs in scorpion venom would be to assist in the digestion of prey. As with ant [33] and spider [47] venoms, cytolytic activity combined with enzymatic activity would help the degradation of the cellular membranes of prey prior to consumption by the scorpion.

## 4. Materials and Methods

### 4.1. Specimen Collection

A permit to collect scorpions was granted by the Wildlife Section, Forestry Division, Trinidad. The study protocol was approved by the University of The West Indies (The UWI) Campus Ethics Committee (CREC-SA.0269/03/2020) and venom was collected by authorized researchers. Female *T. trinitatis* (*n* = 7; head-to-telson length 64–72 mm; weight 1.1–1.9 g) were collected in the Bush Bush Wildlife Sanctuary, Nariva Swamp in May 2022. The scorpions were visualized using a UV light, placed in containers with substrate and adequate ventilation and taken to the Biochemistry Laboratory, The UWI, St. Augustine Campus for venom collection.

### 4.2. Venom Collection

Scorpions were held in captivity for less than 48 h before the venom collection procedure. Venom was obtained via electrostimulation using a stabilized power supply (Farnell Instruments Ltd., Leeds, UK) following the procedures described by Yaqoob et al. [48] and Schaffrath and Predel [49] with minor modifications. Each scorpion was immersed for a few seconds in a saline solution (10% *w*/*v*) to allow greater electrical conductivity followed by restraining with a Velcro tape to a flat base to prevent mobility. The stinger was held with forceps connected directly to one of the electrodes. The second electrode was placed at the third segment of the tail and intermittent 12 V stimuli were applied for 5–10 s until venom expulsion ceased. Each venom sample (approx.10 µL/scorpion) was collected on a metal spatula and mixed with 1% (*v*/*v*) trifluoracetic acid (TFA)/water in a 1:4 ratio. The venom samples were pooled and stored at −20 °C until the time of analysis. The scorpions were released into their habitats on the following day.

### 4.3. Purification of the Peptides

Concentration and partial purification of the peptides in the pooled scorpion venom samples were accomplished by passage through Sep-Pak C-18 cartridges (Waters Associates, Milford, MA, USA) as previously described [50]. Purification to near homogeneity of peptides with antimicrobial activity was accomplished by an initial chromatography on a semipreparative (1.0 cm × 25 cm) Vydac 218TP510 (C-18) reversed-phase HPLC column (Grace, Deerfield, IL, USA) column followed by chromatography of individual components of interest on a Vydac214TP510 (C-4) column. Full details are provided as Supplementary Materials.

### 4.4. Structural Characterization

MALDI-TOF mass spectrometry was carried out using an UltrafleXtreme instrument (Bruker Daltonik, Bremen, Germany). A full description of the procedure, including calibration of the instrument with peptides of known molecular mass in the 1–4 kDa range, has been provided previously [51]. The accuracy of mass determinations was <0.02%. The primary structures of the purified peptides were determined via automated Edman degradation using an Applied Biosystems model 494 Procise sequenator (Applied Biosystems, Courtaboeuf, France). 

The ion-channel toxins present in peaks 1–3 (Figure 1) were identified by CID-MS/MS mass spectrometric analysis of fragments generated by in-gel trypsin digestion as previously described [24]. Full details of the procedure are described in Supplementary Materials.

### 4.5. Peptide Synthesis

TtAP-1, TtAP-2 and TtAP-3 were supplied in crude form by Synpeptide Co., Ltd. (Shanghai, China) and were purified to near homogeneity (>98% purity) via reversed-phase HPLC on a (2.2 cm × 25 cm) Vydac 218TP1022 (C-18) column under the conditions described in Supplementary Materials. The identities of the peptides were confirmed by electrospray mass spectrometry.

### 4.6. Antimicrobial Assays

Antimicrobial activities of synthetic replicates of TtAP-1, TtAP-2 and TtAP-3 were assessed against the following ESKAPE^+^ pathogens: *P. aeruginosa* PA01 DSM 50071, methicillin-resistant *S. aureus* (MRSA) ATCC 43300, *E. coli* DSM 787, vancomycin-resistant *E. faecalis* (VRE) MF06036, *A. baumannii* DSM 30008, carbapenem-resistant *K. pneumoniae* (CRE) ATCC BAA 1705, *S. epidermidis* DSM 28319, vancomycin-resistant *E. faecium* (VRE) NCTC 12201, *C. difficile* strain R20291 DSM 27147 and biofilm-producing *C. difficile* strain 630 ATCC BAA 1382. The MICs for aerobes were determined in duplicate using two separate inocula for each microbe in 96-well microtiter cell-culture plates by a standard double dilution method according to European Standard BS EN ISO 20776-1:2020 guidelines (equivalent to Clinical Laboratory and Standards Institute CLSI M07) [52]. MIC determinations for *C. difficile* strains were performed in a Don Whitley MACS500 anaerobic cabinet according to CLSI M11 guidelines as previously described [53,54]. Control incubations were carried out in parallel with increasing concentrations of gentamicin for all aerobes and vancomycin for *C. difficile*. 

In order to determine the rate at which TtAP-1 caused bacterial cell death, *A. baumannii* (DSM 30008) and *S. aureus* MRSA (ATCC 43300) were selected as test microorganisms. Triplicate overnight cultures of each pathogen were inoculated (1% *v*/*v*) into Mueller Hinton Broth (MHB), grown to an optical density of 0.1 at 625 nm (0.5 McFarland equivalent) and diluted 1/100 in fresh MHB. In a 96-well plate, a 50 µL aliquot of the bacterial inoculum was added to a 50 µL aliquot of peptide TtAP-1 to give a final concentration of peptide of 6.25 µg/mL (1× MIC). Wells were prepared in triplicate for each time point (0, 15, 30, 45, 60 min) with a final well volume of 100 µL. The plate was incubated at 37 °C with 200 rpm orbital shaking. At each time point an aliquot of the relevant well contents was removed and serially diluted 1/10 in Soya Casein Digest Lecithin Polysorbate Broth (SCDLP) prior to spotting 10 µL of each dilution in triplicate onto Mueller–Hinton agar for CFU enumeration. Control incubations in the absence of peptide were carried out.

### 4.7. Hemolysis Assay

Hemolytic activities of the peptides in the concentration range 15.6–250 µg/mL against freshly prepared erythrocytes from male NIH male Swiss mice (Harlan Ltd., Bicester, UK) were determined in duplicate as previously described [50]. The LC_50_ value was taken as the mean concentration of peptide producing 50% hemolysis in three independent incubations.

## 5. Conclusions

The study has identified three peptides (TtAP-1, TtAP-2 and TtAP-3) present in the venom of the scorpion *T. trinitatis* with varying degrees of antimicrobial activity against a range of clinically relevant bacteria. TtAP-1 was the most effective peptide, particularly against ESKAPE^+^ pathogens and *C. difficile*, which represent a major threat to public health. However, the therapeutic potential of the peptide as an agent for the treatment of infections produced by antibiotic-resistant microorganisms is limited by its moderately high hemolytic activity. A further disadvantage of naturally occurring peptides as anti-infective agents, particularly for systemic use, is their rapid clearance following injection. Nevertheless, TtAP-1 represents a template for the design of analogs containing appropriate amino acid substitutions that preserve the high potencies against pathogenic microorganisms while at the same time reducing toxicities against mammalian cells and increasing their half-lives in circulation. Strategies for effecting such transformations in cationic, α- helical peptides such as TtAP-1 are well established and are the subject of recent reviews [55,56,57].

## Figures and Tables

**Table 1:** Identification of toxins present in *T. trinitatis* venom.

Peak No.	Molecular Mass (Da)	Classification	*T. discrepans* Homolog	Accession Number *
1	6986.0	Sodium channel toxin	Toxin TdNa5	C9X4K3.1
2	7280.7	Potassium channel toxin	Toxin TdKIK	Q0GY43.1
2	6957.8	Sodium channel toxin	Toxin TdNa1	C9X4J9.1
3	7682.8	Potassium channel toxin	Toxin Tdi-β-KTx	Q0GY44.1

Peak No. refers to Figure 1. * included in the UniProtKB/Swiss-Prot database.

**Table 2:** Primary structures and molecular masses of the peptides isolated from the venom of female *T. trinitatis*.

Peak No.	Peptide	Primary Structure	[MH^+^]_exp_	[MH^+^]_calc_
4	TtAP-1	FLGSLFSIGSKLLPGVFKLFSRKKQ ^a^	2796.8	2796.6
5	TtAP-2	IFGMIPGLIGGLISAFK ^a^	1733.1	1733.0
6	TtAP-3	FFSLIPSLIGGLVSAIK ^a^	1761.1	1761.1

Peak No. refers to Figure 1. [MH^+^]_exp_ denotes the experimentally determined molecular mass and [MH^+^]_calc_ denotes the mass calculated from the proposed structures. ^a^ denotes C-terminal α-amidation.

**Table 3:** Physicochemical properties of the peptides isolated from *T. triniatis* venom.

Peptide	Charge at pH 7.0	Hydrophobicity	Hydrophobic Moment	Predicted Helical Domain
TtAP-1	+6	0.562	0.427	2–24
TtAP-2	+2	0.906	0.597	4–15
TtAP-3	+2	0.895	0.592	4–15

Mean hydrophobicity was calculated using the hydrophobicity scale of Fauchere et al. [26]. Hydrophobic moment [27], a measure of the amphipathicity of an α-helix, was calculated using the HeliQuest web server [28]. Helical domains were predicted using the PredictProtein program [29].

**Table 4:** Minimum inhibitory concentrations (µg/mL) for peptides isolated from *T. trinitatis* venom against a range of clinically relevant Gram-positive and Gram-negative microbial pathogens.

Microorganism	Peptide TtAP-1	Peptide TtAP-2	Peptide TtAP-3	GEN	VAN
*P. aeruginosa* DSM 50071	12.5	>400	>400	3.91	nd
*E. coli* DSM 787	6.25	100	200	0.98	nd
*K. pneumoniae* ATCC BAA 1705	6.25	>100	>400	0.98	nd
*A. baumannii* DSM 30008	6.25	50	100	0.98	nd
*S. aureus* ATCC 43300	6.25	25	25	31.3	nd
*S. epidermidis* DSM 28319	6.25	25	100	0.49	nd
*E. faecalis* MF 06036	6.25	50	100	3.91	nd
*E. faecium* NCTC 12201	6.25	25	50	1.95	nd
*C. difficile* DSM 27147	3.13	25	50	nd	1.0
*C. difficile* ATCC BAA 1382	3.13	25	50	nd	1.0

nd: not determined, GEN: gentamicin, VAN: vancomycin.

## Data Availability

The data presented in this study are available on request from the corresponding author.

## Supplementary Materials

The following supporting information can be downloaded at: https://www.mdpi.com/article/10.3390/antibiotics12091404/s1, File S1: Methods.
