# Supplementary material for: Identification of an Antimicrobial Peptide from the Venom of the Trinidad Thick-Tailed Scorpion Tityus trinitatis with Potent Activity against ESKAPE Pathogens and Clostridioides difficile
Source: Antibiotics (Basel). 2023 Sep 4;12(9):1404. doi: 10.3390/antibiotics12091404 (PMC10525828; doi:10.3390/antibiotics12091404)
Supplement: Supplementary file 1 [file antibiotics-12-01404-s001.zip › antibiotics-2574053-supplementary.pdf]

antibiotics

Supplementary Materials

Purification of the peptides

The pooled scorpion venom samples were diluted with 0.1% (v/v) TFA/water (50 mL) and concentration and partial purification of the peptides was accomplished by passage at a flow rate of approximately 2 mL.min^{-1} through two Sep-Pak C-18 cartridges connected in series (Waters Associates, Milford, MA, USA) as previously described [50]. Bound material was eluted with acetonitrile/water/TFA (70.0:29.9:0.1, v/v/v) and freeze-dried. The material was redissolved in 0.1% (v/v) TFA/water (2 mL) and injected onto a semipreparative (1.0 cm x 25 cm) Vydac 218TP510 (C-18) reversed-phase HPLC column (Grace, Deerfield, IL, USA) equilibrated with 0.1% (v/v) TFA/water at a flow rate of 2.0 mL.min^{-1}. The concentration of acetonitrile in the eluting solvent was raised to 21% (v/v) over 10 min and to 63% (v/v) over 60 min using linear gradients. Absorbance was monitored at 214 nm and fractions (1 min) were collected using a BioRad 2110 fraction collector. The peptides within the peaks designated 4 – 6 (Figure 1) containing the antimicrobial peptides were subjected to further purification by chromatography on (1.0 cm x 25 cm) Vydac 214TP510 (C-4) column. The concentration of acetonitrile in the eluting solvent was raised from 21% to 56% (v/v) over 50 min. The flow rate was 2.0 mL.min^{-1} and fractions were collected by hand. The peaks 1 - 3 containing the ion-channel toxins were purified on the same column. The concentration of acetonitrile in the eluting solvent was raised from 21% to 42% (v/v) over 50 min and the flow rate was 2.0 mL.min^{-1}.

Peptide synthesis

TtAP-1, TtAP-2 and TtAP-3 were supplied in crude form by Synpeptide Co., Ltd. (Shanghai, China). They were purified to near homogeneity (> 98% purity), assessed by symmetrical peak shape and mass spectrometry, by reversed-phase HPLC on a (2.2 cm x 25 cm) Vydac 218TP1022 (C-18) column equilibrated with acetonitrile/water/TFA (35.0/64.9.9.9/0.1, v/v/v) at a flow rate of 6 mL.min^{-1}. The concentration of acetonitrile was raised to 63% (v/v) over 60 min using a linear gradient. Absorbance was measured at 214 nm and the major peak in the chromatogram was collected manually. The identities of the peptides were confirmed by electrospray mass spectrometry.

Proteomic analysis of the purified toxins

The molecular masses of the purified components were determined by nano-Acquity Ultra Performance LC (Waters Corporation, Milford, MA, USA) using a BEH130 C-18 (100 µm x 100 mm, 1.7 µm particle size) column in-line with a Waters SYNAPT G2 High Definition Mass Spectrometry System. The flow rate was set to 0.6 µL.min^{-1} and the column was developed with a linear gradient of 0.1% formic acid in water (solution A) and 0.1% formic acid in acetonitrile (solution B), isocratically 1% B for 1 min, followed by 1–12% B for 1 min, 12–40% B for 15 min, 40–85% B for 2 min. Monoisotopic molecular masses were calculated by manual deconvolution of the isotope-resolved multiply charged MS1 mass spectra. The purified toxins were initially analysed by SDS-PAGE on 15% polyacrylamide gels under reducing and non-reducing conditions and the protein bands were excised from Coomassie Brilliant Blue-stained gels and subjected to automated reduction, alkylation, and in-gel digestion with sequencing grade porcine pancreatic trypsin using a Progest™ digestor (Genomic Solutions, Ann Arbor, MI, USA). Tryptic digests were dried in a SpeedVac vacuum centrifuge, redissolved in 16 µL of 0.1% formic acid in water and submitted to LCMS/MS. Tryptic peptides were separated by nano-Acquity Ultra Performance LC as described above. Doubly and triply charged ions were selected for collision-induced dissociation (CID)-MS/MS. Fragmentation spectra were interpreted (a) manually (de novo sequencing), (b) using the on-line form of the MASCOT Server (version 2.8.1.242) at http://www.matrixscience.com against the last update (December 7th, 2022) of the NCBI non-redundant database, and (c) processed in the Waters Corporation's ProteinLynx Global SERVER 2013 version 2.5.2. (with Expression version 2.0). The following search parameters were used: Taxonomy: Metazoa (Animals); Enzyme: trypsin (two missed cleavage allowed); MS/MS mass tolerance was set to ± 0.6 Da; carbamidomethyl cysteine and oxidation of methionine were selected as fixed and variable modifications, respectively. All matched MS/MS data were manually checked. Peptide sequences assigned by de novo MS/MS were matched to homologous proteins available in the NCBI non-redundant protein sequences database using the online BLASTP program at https://blast.ncbi.nlm.nih.gov/Blast.cgi.
